# Dialysis decision-making process by Chinese American patients at an urban, academic medical center: a retrospective chart review

**DOI:** 10.1186/s12904-024-01357-y

**Published:** 2024-01-25

**Authors:** Abigail L. Lebovitz, Steven A. Schwab, Michelle M. Richardson, Klemens B. Meyer, Benjamin Sweigart, Tamara Vesel

**Affiliations:** 1 Tufts University School of Medicine, Boston, MA USA; 2 Tufts Medical Center, Department of Medicine, William B. Schwartz Division of Nephrology, Tufts University School of Medicine, Dialysis Clinic, Inc., Boston, MA USA; 3 Tufts Clinical and Translational Science Institute, Boston, MA USA; 4 Tufts Medical Center, Department of Medicine, Division of Palliative Care, Tufts University School of Medicine, 800 Washington Street, Boston, MA 02111 USA

**Keywords:** Decision-making, Chinese American, Chronic kidney disease, Kidney failure, Chart review

## Abstract

**Background:**

Clinical practice guidelines emphasize shared decision-making for kidney replacement treatment, yet little is known about the influence of cultural differences on that process. We undertook a retrospective chart review to explore the process and timing of dialysis decision making and initiation in Chinese American patients to provide quality kidney care for this population.

**Design:**

Participants received outpatient care at Tufts Medical Center and dialysis at Dialysis Clinic, Inc. Boston or Somerville, MA from 2001–2021. Clinic chart review sourced demographic, clinical, and end-of-life care information from 180 participants (82 Chinese American, 98 other) from stage 4 chronic kidney disease (CKD) and dialysis initiation.

**Results:**

Chinese American participants were older (mean 70 vs. 59, *p* < 0.0001), less likely to speak English (12% vs. 87%, *p* < 0.0001), and used interpreter services more (80% vs. 11%, *p* < 0.0001). Chinese American participants had more visits (median 14 vs. 10, *p* = 0.005); were more often accompanied by family members (75% vs. 40%, *p* < 0.001); and had significantly lower rates of healthcare proxy documentation (35% vs. 55%, *p* = 0.006). There was no statistical difference in months between first CKD 4 visit and first dialysis. Both groups started dialysis at the same average eGFR and with similar rates of permanent dialysis access. Chinese American participants had significantly lower serum albumin at dialysis initiation (mean 3.3 g/dL vs 3.7 g/dL, *p* = 0.0003). Documentation reflected a low number of conversations about non-dialytic care, end-of-life planning, or palliative care in both groups across all visits.

**Conclusion:**

The time between CKD 4 and dialysis initiation was the same in both groups, suggesting a similar overall outcome of care. Chart documentation suggests that Chinese American participants had a significantly higher number of visits with nephrologists where discussion about dialysis was noted and were more likely to have a family member present at the visit. Fewer Chinese American participants completed healthcare proxies. Among all study participants, healthcare proxy, code status, and palliative care discussions were reported less frequently than expected. These findings highlight opportunities for collaboration between palliative care clinicians and nephrologists.

**Supplementary Information:**

The online version contains supplementary material available at 10.1186/s12904-024-01357-y.

## Introduction

Chronic kidney disease (CKD) is one of the leading noncommunicable causes of death across the world, and is associated with significant morbidity, low quality of life, and progression to kidney failure [1–4]. As the population ages and treatment of comorbid conditions becomes more effective, more individuals will survive to reach the decision whether and when to use dialysis to reduce uremic symptoms and to extend their lives.

Current international clinical practice guidelines on kidney failure management and other recommendations to improve quality of care emphasize a shared decision-making process to help patients make a timely decision that aligns with their values and preferences [5–9]. Shared decision-making has been defined as an “approach where clinicians and patients make decisions together using the best available evidence” [10]. Literature in other chronic disease states suggests that cultural and linguistic differences can complicate communication and shared decision-making between patients and providers [6, 11, 12]. Better understanding of cultural variations in behavior and their underlying psychological and social causes can make shared decision-making more effective through culturally appropriate communication [13]. However, kidney-specific recommendations have yet to formalize how kidney care providers should identify the influences of cultures and integrate them into shared decision-making [5–9].

Tufts Medical Center is an academic medical center located in Boston’s Chinatown neighborhood. Approximately 10% of all patients receiving care at Tufts Medical Center are Chinese American [14, 15] and approximately 22% of all patients in the on-site dialysis clinic operated by Dialysis Clinic, Inc. (DCI) are Chinese American. We undertook a retrospective chart review to explore the process and timing of dialysis decision making and initiation in Chinese American patients to provide quality kidney care for this population.

## Methods

Participants were identified by one author (KBM) from an electronic health database of all patients who received outpatient CKD care at Tufts Medical Center and who began dialysis at DCI between January 2005, when automated reporting of estimated glomerular filtration rate (eGFR) began at Tufts Medical Center, and June 2021.

We collected and reported GFR as estimated by the CKD-EPI equation and subsequently by the CKD-EPI 2009 [16] equation because these equations represented the standard of care for reporting of eGFR at the time of the care on which we are reporting. Those equations contain a coefficient for race; Chinese Americans would have been classified as non-African American. More recently, a National Kidney Foundation and American Society of Nephrology Joint Task Force has recommended use of the CKD-EPI 2021 equation, which does not contain a race coefficient [17]. Data were obtained from electronic health records: eClinicalWorks™ for outpatient records and Soarian™ for inpatient records and for participant demographics. Two medical students and investigators (ALL, SAS) completed data collection using a standardized data collection form developed by the research team. ALL and SAS had no prior exposure to the nephrology clinic and dialysis facility patient population before data abstraction. They received training on how to review charts objectively and were trained on CKD and dialysis-related language that was unique to this clinical situation. They were not blinded to the research question when they abstracted data. Data were managed using Research Electronic Data Capture (REDCap) hosted at Tufts University [18, 19]. To ensure inter-rater reliability, data collection was duplicated in five percent of records.

We collected data at three pre-defined time periods: 1) the first nephrology clinic visit when the participant’s eGFR was less than 30 ml/min/1.73m^2^, hereto referred to as “first CKD 4 visit”; 2) the time period between the first CKD 4 visit and the date of first of dialysis, hereto referred to as “time from CKD 4 to first dialysis”; and 3) the date of first dialysis, hereto referred to as “first dialysis”. These three time periods were defined to examine the time between CKD 4 and dialysis initiation, as this is a critical period in nephrology care when providers are more likely to begin counseling regarding kidney failure and the need for dialysis. Collected data elements included: demographic characteristics, clinical laboratory variables routinely used in CKD and dialysis care [20] estimated glomerular filtration rate, visit characteristics, discussions of kidney failure treatment and documentation of palliative care and end-of-life planning.

Self-reported race and ethnicity were extracted from Soarian™. For this study, Chinese American refers to any adult (> 18 years old) living in the United States who self-identified as ethnically Chinese, regardless of birth country [21]. Any participant who had not self-identified as Chinese American in the electronic health record was categorized as “other.”

We compared group characteristics using chi-squared and Fisher’s exact tests for categorical variables and t-tests or Wilcoxon rank-sum tests for continuous variables. We constructed exploratory multivariable regression models to assess the potential association between ethnicity and outcomes of interest. We modeled time from first CKD 4 visit to first dialysis using linear regression with a log-transformed outcome. We modeled the number of visits after first CKD 4 visit until first dialysis using negative binomial regression, with log-transformed time from first CKD 4 visit to first dialysis as an offset. Models included sex, age, and potential confounders chosen on the basis of known relationship with biological health in kidney disease. Variables included in adjusted analysis were not chosen a priori and further analysis were included for exploratory purposes.

For all analyses a two-sided 0.01 level of significance was set in advance due to the number of statistical tests performed. All analyses were conducted using SAS Enterprise Guide software, Version 8.3 Update 3 (SAS Institute Inc., Cary, NC).

Study results were shared with a community advisory board consisting of 5 members selected by our community partner. The members ranged in age from 25 to 75 years of age; 80% were women; and all spoke English. The advisory board was developed to provide feedback, nuance and interpretation of study results and ensured that the research team approached the study and its results in a culturally respectful manner.

## Results

The initial cohort included 227 participants who received both outpatient nephrology care at Tufts MC and who began dialysis at DCI between January 2005 and June 2021. 47 participants were excluded either because they received nephrology care at Tufts MC before dialysis, but after the onset of CKD stage 4 and or had fewer than three clinic visits with a Tufts MC nephrologist. Thus, we identified 180 participants (82 Chinese American, 98 other) with a first CKD 4 visit who received outpatient CKD care at Tufts Medical Center and had their first dialysis at DCI during the study period.

Table 1 shows that the two groups were similar at the first CKD 4 visit with a few notable exceptions. Chinese American patients were significantly older (*p* < 0.0001) than other patients; were less likely to speak English (*p* < 0.0001); and used interpreter services more often (*p* < 0.001). Chinese American participants had religion recorded significantly less frequently in the electronic health record (*p* < 0.0001) and had significantly fewer completed health care proxy documents (*p* = 0.006). Comorbid conditions and laboratory results were similar across both groups with the exception that Chinese American participants were significantly less likely to have received a prior kidney transplant (*p* < 0.001) and significantly less likely to have received dialysis in the past (*p* = 0.004). Finally, Chinese Americans had lower diastolic blood pressure values (*p* = 0.002) and parathyroid hormone concentrations (*p* < 0.001) (see Supplemental Table 1).

**Table 1:** Participant characteristics at first CKD 4 visit

	**All (*N* = 180)**	**Chinese American (*N* = 82)**	**Others (*N* = 98)**	***p*-value**
Female, n (%)	66 (36.7)	27 (32.9)	39 (39.8)	0.34
Age, mean (SD)	64.1 (13.9) [*N* = 174]	69.8 (10.9) [*N* = 81]	59.1 (14.4) [*N* = 93]	< 0.0001
Race, n (%)				
White			45 (45.9)	
Black			30 (30.6)	
Hispanic			9 (9.2)	
Other			14 (14.3)	
Language spoken, n (%)				
English	95 (52.8)	10 (12.2)	85 (86.7)	< 0.0001
Cantonese	62 (34.4)	61 (74.4)	1 (1.0)	< 0.0001
Mandarin	6 (3.3)	6 (7.3)	0 (0.0)	0.008
Spanish	4 (2.2)	0 (0.0)	4 (4.1)	0.13
Vietnamese	7 (3.9)	0 (0.0)	7 (7.1)	0.016
Other	9 (5.0)	7 (8.5)	2 (2.0)	0.08
Interpreter required, n (%)	73 (41.5)	62 (79.5)	11 (11.2)	< 0.0001
Religion reported, n (%)	106 (58.9)	28 (34.2)	78 (79.6)	< 0.0001
Hypertension, n (%)	162 (91.5)	76 (92.7)	86 (90.5)	0.61
Prior kidney transplant, n (%)	19 (11.0)	2 (2.6)	17 (17.9)	0.0013
Prior dialysis treatment, n (%)	20 (11.7)	3 (3.9)	17 (18.1)	0.004
Healthcare proxy completed, n (%)	81 (45.8)	28 (34.6)	53 (55.2)	0.006

Table 2 displays visit and clinical characteristics for the time between first CKD 4 visit and the first dialysis. During that time, Chinese American participants had a significantly higher number of visits (*p* < 0.005). However, there was no significant difference in time elapsed in months or the rate of visits per year. Chinese American participants were more often accompanied by a family member (*p* < 0.0001), and there were more physician telephone conversations with family members in the absence of the patient in the Chinese American group (*p* = 0.006). Clinical characteristics were similar between groups with the exception that Chinese American participants reported itching significantly more often (*p* < 0.0001) (see Supplemental Table 2).

**Table 2:** Participant characteristics between first CKD 4 visit and first dialysis

	**All (*N* = 180)**	**Chinese American (*N* = 82)**	**Others (*N* = 98)**	***P*-value**
Time between first CKD 4 visit and first dialysis (months), median (IQR)	22.3 (10.5–39.5)	24.6 (13.6–47.5)	21.2 (9.3–37.3)	0.19
Number of visits between first CKD 4 visit and first dialysis, median (IQR)	12 (6–20)	14 (8–22)	10 (4–17)	0.005
Number of visits per year between first CKD 4 visit and first dialysis, median (IQR)	6.6 (4.6–10.3)	7.2 (4.9–11.1)	6.0 (4.3–9.1)	0.14
Dialysis was addressed, n (%)	174 (99.4)	80 (100.0)	94 (99.0)	1.00
Number of times dialysis documented in clinic notes, median (IQR)	7 (4–11)	9 (5–14)	6 (4–10)	0.016
Prognosis was addressed, n (%)	54 (33.1)	26 (34.2)	28 (32.2)	0.78
Interpreter present, n (%)	61 (35.9)	50 (67.7)	11 (11.6)	< 0.0001
Professional interpreter, n (%)	47 (26.1)	42 (51.2)	5 (5.1)	< 0.0001
Family member, n (%)	28 (15.6)	22 (26.8)	6 (6.1)	< 0.0001
Family member was present at visit, n (%)	98 (56.0)	60 (75.0)	38 (40.0)	< 0.0001
Participants with understanding of treatment documented in electronic health record, n (%)	43 (30.9)	25 (38.5)	18 (24.3)	0.07
Alternative to dialysis treatment addressed in visit, n (%)	93 (53.8)	39 (49.4)	54 (57.5)	0.29
Transplant, n (%)	75 (41.7)	27 (32.9)	48 (49.0)	0.03
Conservative care, n (%)	16 (8.9)	8 (9.8)	8 (8.2)	0.71
Symptom management / palliative care / end-of-life care, n (%)	10 (5.6)	6 (7.3)	4 (4.1)	0.35
Any conversations between provider and proxy/family, n (%)	23 (14.8)	16 (23.9)	7 (8.0)	0.006
Dialysis as a bridge to transplant noted, n (%)	35 (24.3)	12 (17.1)	23 (31.1)	0.05

Table 3 displays data at the time of the first dialysis. Average eGFR at dialysis initiation was 10 mL/min/1.73m^2^ in both groups. Chinese American participants were older at first dialysis (*p* < 0.0001). There were low rates (18%) of code status reported for all participants with no significant difference across groups. Clinical data at first dialysis (Supplemental Table 3) were similar between groups with the exception of serum albumin concentration being significantly lower in Chinese American participants (*p* = 0.0003).

**Table 3:** Participant characteristics at first dialysis

	**All (*N* = 180)**	**Chinese American (*N* = 82)**	**Others (*N* = 98)**	***P*-value**
Age, mean (SD)	66.5 (14.6)	72.7 (11.4)	61.0 (14.9)	< 0.0001
eGFR (mL/min/1.73m2), mean (SD)	9.9 (4.5)	9.6 (5.1)	10.1 (3.9)	0.50
Arteriovenous fistula or graft placed prior to first dialysis, n (%)	106 (61.3)	48 (61.3)	58 (61.1)	0.95
Peritoneal dialysis catheter placed prior to first dialysis, n (%)	47 (27.0)	16 (20.0)	31 (33.0)	0.05
Transplant candidate, n (%)	35 (20.0)	14 (17.3)	21 (22.3)	0.40
First dialysis initiated in hospital, n (%)	98 (65.3)	49 (73.1)	49 (59.0)	0.07
Code status reported, n (%)	33 (18.3)	10 (12.2)	23 (23.5)	0.05

To explore possible relationships among variables and between groups, we undertook limited hypothesis-generating multivariable comparisons. Univariate analysis found that Chinese American participants had more outpatient visits between first CKD 4 visit and first dialysis. This finding remained significant (*p* < 0.0001) when adjusting for age at first CKD 4 visit, eGFR, sex, and serum albumin concentration at first CKD 4 visit (see Table 4).

**Table 4:** Negative binomial regression of number of visits between first CKD 4 visit and first dialysis

Variable	Ratio of Counts	95% Confidence Limits		*p*-value
Chinese American	1.61	1.27	2.03	< 0.0001
Age at first CKD 4 visit	1.00	0.99	1.00	0.292
Value of eGFR < 30 mL/min/1.73m^2^	1.06	1.04	1.08	< 0.0001
Female	1.21	0.96	1.52	0.109
Serum albumin at first CKD 4 visit (g/dL)	1.03^a^	1.01	1.05	0.0011

^a^Effect of a 0.1-unit change in serum albumin

In univariate analysis, there was no statistically significant difference between groups in the amount of time to start dialysis from CKD stage 4. The association remained non-significant after adjusting for age at first CKD 4 visit and for sex (see Supplemental Table 4). Additionally, the rate of visits per year remained non-significant after adjusting for age and sex (see Supplemental Table 5).

Univariate analysis found that Chinese American participants had lower serum albumin concentration at first dialysis. When adjusting for age, there was a trend to lower albumin concentration among Chinese American participants at first dialysis; however, it was not a significant difference (*p* < 0.0126) based on our predetermined significance level (see Table 5).

**Table 5:** Linear regression of serum albumin concentration at first

Variable	Estimate	Standard Error	*p* Value
Intercept	4.34	0.26	< 0.001
Chinese American	-0.29	0.11	0.0126
Age at first dialysis	-0.01	0.00	0.0098

## Discussion

Our chart review results refuted our initial hypothesis: we found no difference in time from first CKD 4 visit to first dialysis between Chinese American patients and others in our nephrology clinic. However, we did find differences in group characteristics at all three pre-defined data collection time periods. At first CKD 4 visit, Chinese American participants were older, less likely to speak English and as a result, more likely to use interpreter services. Chinese American participants had significantly fewer completed health care proxy documents and had significantly more visits between first CKD 4 visit and first dialysis. However, the time to the first dialysis and the rate of visits were not significantly different between groups.

Although kidney failure is a life-threatening illness with high mortality rates, low quality of life and significant symptom and treatment burden [1–3], we found overall low rates of symptom, end-of-life care, or palliative care discussions during the time between first CKD 4 visit and first dialysis. Only 10% of all participants had documented conversations about end-of-life care decisions and18% had resuscitation wishes specified through code status. One explanation may be that nephrologists develop relationships with patients during the multiple visits leading up to dialysis initiation and may be hesitant to discuss end-of-life care for fear of taking hope away from patients. Christakis et al. and Gerber et al. describe the phenomenon that as duration of patient-doctor relation increases and time since last contact decreases, prognostic accuracy decreases [22, 23]. Patients may also avoid end-of-life conversations, and, thus, clinicians may choose to emphasize life prolonging options, such as dialysis. Additionally, it is possible that these sensitive conversations occur between provider and patient, but are not being documented in the medical record [24]. Literature examining concordance between observed clinical encounters and electronic health records suggests that documentation may not fully reflect what occurs in the clinical encounter [25, 26]. Further study is needed to better understand any discrepancies between end-of-life discussions during a visit discussing kidney failure treatment as compared to what is documented in a patient’s electronic health record.

Low rates of end-of-life or palliative care discussion in this study suggests an area of further collaboration between nephrologists and palliative care physicians. Literature acknowledges the unmet palliative care needs of patients with CKD and the barriers to palliative care within nephrology [27]. Despite these unmet needs, proactive and early integration of palliative care in the treatment of individuals with CKD improves patient outcomes [28]. At Tufts Medical Center, this study and further educational and research collaboration between nephrologists and palliative care physicians have contributed to the development of formal palliative care consultation for patients with CKD 4 and 5. Treatment of CKD 4 and 5 in a setting in which kidney function estimation is performed routinely offers patients the opportunity to plan ahead and to make or defer decisions regarding treatment, whether transplantation, dialysis or non-dialytic medical management. Dialysis treatment can extend life by decades for some patients, and by months or years for others. However, in older patients, survival or quality of life may not benefit from dialysis [9]. Our study found no difference across study groups in documented conversations about alternatives to dialysis, including conversations regarding transplant, conservative care (non-dialytic, non-transplant supportive care) and palliative care. Only 9% of all participants in our study had any documented conversations about conservative treatment of kidney failure. The acceptance of this approach to kidney failure management as a routine alternative to dialysis has increased in recent years, and our 16-year sample, may underestimate the current offering of this treatment option [13].

Additionally, literature shows that Chinese American families emphasize harmony and mutual reliance within the family [14] and a strong sense of filial piety [6, 7]. These factors may move discussion away from consideration of non-dialytic care and towards dialysis. Dialysis may be seen as a more active treatment that fulfills responsibilities arising from filial piety. However, the limited detail available in the records did not allow us to elucidate the specific role of filial piety, this attribute has been found to be associated with a preference for active medical management among Chinese families [29–31]. Chinese culture emphasizes respect for physicians [14], and it is possible that nephrologists can help families by framing treatment decision discussions to suggest that any kidney failure treatment choice can fulfill familial obligations (*e.g.*, respecting patient’s wishes, offering additional support for care at home).

Language barriers may also be important when assessing dialysis decision making within this population, with changes in meaning and tone during interpretation of conversations with patients even when performed by certified medical interpreters. Such subtle effects may influence patient and family understanding and decision making [32].

We noted that there was a low proportion of advance care planning markers, such as health care proxy and desire for resuscitation among Chinese American participants. Despite the average healthcare proxy documentation for the total study population being less than half of the participants (46%), these numbers are higher than the national level [33]. Additionally, a family member was present for at least one visit for 76% of Chinese American participants. Perhaps, this is a missed opportunity to identify health care proxies and facilitate goals of care conversations. The role of family member presence in facilitating conversation about advanced care planning needs to be further explored; there may be cultural differences in familial duty and protection of family members from sensitive information. These dynamics may trump the conversation around end of life in Chinese American patients [34–39]. Our findings of more visits with family present and more telephone calls with family members could be used as opportunities to identify the family hierarchy and how it overlaps with documentation of health care proxies, complete advance care planning documents, and build trusting relationships.

Regarding clinical comparisons, the study groups differed in age, language spoken, the need for interpreter services and the likelihood of having a previous kidney transplant at first CKD 4 visit. These differences may reflect the location of Tufts Medical Center in Boston’s Chinatown community. Tufts Medical Center serves as a community hospital for Chinatown residents, whereas patients from a larger catchment area seek tertiary specialty care.

Between first CKD 4 visit and first dialysis, Chinese American participants had more clinic visits, however this relationship was no longer statistically significant when multivariable analyses were applied. The time between first CKD 4 visit and first dialysis and the rate of visits per year in this time period were similar in both groups; they started dialysis at the same average eGFR and had similar rates of permanent dialysis access at dialysis initiation which suggests parity in care between groups.

From the clinician’s point of view, delaying the initiation of dialysis treatment, if it can be done safely, and beginning dialysis with permanent access are highly desirable. Based on the observations about time to dialysis, eGFR value, and access at dialysis initiation, our chart review suggests that Chinese American patients at Tufts Medical Center received care equivalent to other participants. Literature suggests that patients with more pre-dialysis visits had better peritoneal dialysis-related outcomes, fewer cardiovascular events and lower mortality [40, 41].

A detailed look at the cohort characteristics at the first dialysis showed a trend toward more inpatient dialysis initiation and fewer peritoneal dialysis catheters placed in Chinese American participants. Our study is not able to answer why these trends exist, but they highlight additional areas for assessment and potential improvement. Our questions include: Is it patient or clinician preference to start dialysis in the hospital? Is the decision related to the need for interpreters or the perceived need for an interpreter to safely initiate dialysis? Are Chinese Americans offered the option of home dialysis less often, or do they choose home dialysis less often?

Clinical laboratory data show Chinese American participants to be older and to have had significantly lower serum albumin concentration at first dialysis. Serum albumin is a known predictor of overall health in patients with CKD and those on dialysis and is on marker used by nephrologists for prognostication and dialysis initiation timing. There is a known relationship between low serum albumin concentrations and worse survival among dialysis patients [42] and individuals with CKD [43]. It is also known that aging is associated with lower serum albumin concentrations; the older the patient and the lower the albumin concentration the higher risk of morbidity and mortality [44]. Although, adjusted for age, the serum albumin concentration difference, is of borderline significance *p*-value (*p* < 0.0126), our data show that Chinese American patients to have lower serum albumin concentration in each age group. Therefore, the difference in albumin concentrations cannot be fully explained by Chinese American participants being older at the date of first dialysis. This finding may suggest that in our study population, Chinese American patients are more vulnerable when they begin dialysis. Nutrition is another variable that is known to influence serum albumin concentration; however, we did not explore nutritional aspect of lower albumin concentrations in our study.

The strengths of this study include our large population of self-identified Chinese American patients and being able to compare the decision-making process to start dialysis by those participants compared to others. In most studies, patients are categorized more broadly by race (*i.e.*, Asian), which may obscure the specific decision-making considerations within Asian ethnicities and make assumptions that the individual ethnic cultural considerations are the same. Another strength was the close relationship between Tufts Medical Center and DCI that allows for a seamless care experience between patients with kidney failure treated at Tufts who them progress to needing dialysis in their community provided by DCI. This close relationship allowed for us to describe the trajectory from the first CKD 4 visit to the start of dialysis.

The main limitation of the study is that based on our aim, we examined records only of patients who began dialysis, not of those who chose non-dialytic management of kidney failure or died prior to reaching first dialysis. Thus, we are unable to fully describe our entire population and decision-making process around conservative management as a treatment choice. A source of bias included that data abstractors were not blinded to the research questions; however, the two abstractors had no prior experience within the nephrology clinic and no interaction with research participants. Additional limitations include a sixteen-year time frame of patient care to achieve adequate sample size of Chinese American participants, during which there may have been secular changes in patient and clinician behaviors and documentation style. Electronic health records do not contain the full description of patient encounters, and important conversations may not have been documented. Additionally, data may be missing or incorrect in the chart; this can be exacerbated when using interpreters for non-English speaking patients [45].

## Conclusions

This study is unique in that it aimed to explore the decision-making process regarding dialysis initiation with a specific focus on Chinese American patients via a chart review approach. Our study found that both groups meet clinical practice guidelines for the appropriate start of dialysis despite ethnicity, language, and cultural differences. Importantly, these findings highlight the need to enhance palliative care interventions within nephrology care to support patients who are facing the choice of treatment for kidney failure. Intervention may include integration of palliative care physicians into the nephrology clinic or processes to encourage palliative care referral earlier in disease course. Additional exploration is needed to further elucidate how Chinese American patients’ cultural beliefs and attitudes influence interactions with Western medical care and ultimately shared medical decision-making about dialysis.

### Supplementary Information

**Additional file 1: Supplemental Table 1.** Additional participant characteristics and clinical data at first CKD 4 visit. **Supplemental Table 2.** Additional data at time between first CKD 4 visit and dialysis. **Supplemental Table 3.** Additional clinical data at time of first dialysis. **Supplemental Table 4.** Additional linear regression of log-transformed time between first CKD 4 visit and first dialysis. **Supplemental Table 5.** Additional negative binomial regression of rate of visits between first CKD 4 visit and first dialysis.

## Data Availability

Data are available at the request of the author.
