# Supplementary material for: Dialysis decision-making process by Chinese American patients at an urban, academic medical center: a retrospective chart review
Source: BMC Palliat Care. 2024 Jan 25;23:25. doi: 10.1186/s12904-024-01357-y (PMC10809624; doi:10.1186/s12904-024-01357-y)
Supplement: Supplementary file 1 — Additional file 1: Supplemental Table 1. Additional participant characteristics and clinical data at first CKD 4 visit. Supplemental Table 2. Additional data at time between first CKD 4 visit and dialysis. Supplemental Table 3. Additional clinical data at time of first dialysis. Supplemental Table 4. Additional linear regression of log-transformed time between first CKD 4 visit and first dialysis. Supplemental Table 5. Additional negative binomial regression of rate of visits between first CKD 4 visit and first dialysis. [file 12904_2024_1357_MOESM1_ESM.docx]

Supplemental Table 1: Participant Characteristics and Clinical Data at first CKD 4 visit

|  | **Total**  **(N=180)** | **Chinese American**  **(N=82)** | **Others (N=98)** | **p-value** |
| --- | --- | --- | --- | --- |
| Diabetes, n (%) | 87 (49.7)  [N=175] | 42 (51.2) | 45 (48.4)  [N=93] | 0.71 |
| History of heart disease, n (%) | 74 (42.1)  [N=176] | 26 (32.1) [N=81] | 48 (50.5) [N=95] | 0.014 |
| History of malignancy, n (%) | 38 (21.7)  [N=175] | 13 (16.1) [N=81] | 25 (27.2) [N=94] | 0.09 |
| Family history of kidney disease, n (%) | 22 (17.9)  [N=123] | 7 (13.7) [N=51] | 15 (20.8) [N=72] | 0.31 |
| Family history of dialysis, n (%) | 15 (12.6)  [N=119] | 3 (6.3) [N=48] | 12 (16.9) [N=71] | 0.09 |
| eGFR, mean (SD) | 22.5 (5.8)  [N=174] | 22.6 (5.8) [N=80] | 22.5 (5.9) [N=94] | 0.91 |
| Systolic Blood Pressure, mean (SD) | 140.7 (24.1)  [N=168] | 139.3 (22.6) [N=76] | 141.9 (25.4) [N=92] | 0.49 |
| Diastolic Blood Pressure, mean (SD) | 75.1 (13.5)  [N=168] | 71.6 (11.6) [N=76] | 78.1 (14.4) [N=92] | 0.002 |
| Calcium, mean (SD) | 9.0 (0.6)  [N=154] | 9.0 (0.6) [N=73] | 9.0 (0.7) [N=81] | 0.75 |
| PTH, median [Q1-Q3] | 162 [77-276]  [N=90] | 112 [60-171] [N=41] | 210 [121-326] [N=49] | 0.001 |
| Serum albumin, mean (SD) | 3.5 (0.6)  [N=140] | 3.5 (0.7) [N=68] | 3.5 (0.6) [N=72] | 0.76 |
| Phosphorous, mean (SD) | 4.1 (0.9)  [N=149] | 4.1 (0.9) [N=71] | 4.0 (0.9) [N=78] | 0.80 |
| BUN, mean (SD) | 46.6 (15.7)  [N=160] | 49.3 (17.3) [N=76] | 44.1 (13.7) [N=84] | 0.04 |
| Hemoglobin, mean (SD) | 11.2 (1.7)  [N=135] | 11.4 (1.5) [N=57] | 11.0 (1.9) [N=78] | 0.24 |
| Bicarbonate, mean (SD) | 23.3 (3.4)  [N=171] | 24.0 (3.6) [N=79] | 22.8 (3.2) [N=92] | 0.02 |
| Potassium, median [Q1-Q3] | 4.5 [4.2-4.9]  [N=169] | 4.5 [4.1-4.8] [N=79] | 4.5 [4.2-4.9] [N=90] | 0.78 |
| Creatinine, mean (SD) | 3.0 (1.1)  [N=170] | 2.9 (0.9) [N=78] | 3.1 (1.3) [N=92] | 0.28 |

Supplemental Table 2: Additional data at time between first CKD 4 visit and dialysis

|  | **Total**  **(N=180)** | **Chinese American**  **(N=82)** | **Others**  **(N=98)** | **P-value** |
| --- | --- | --- | --- | --- |
| Number of times dialysis documentation in clinic notes (novel), median (IQR) | 7 (4-9) | 7 (4-10) | 5 (3-8) | 0.034 |
| Interpreter present |  |  |  |  |
| Nurse, n (%) | 1 (0.6) | 0 (0.0) | 1 (1.0) | 1.00 |
| Other, n (%) | 2 (1.1) | 0 (0.0) | 2 (2.0) | 0.5 |
| Symptoms recorded |  |  |  |  |
| Fatigue, n (%) | 130 (72.2) | 62 (75.6) | 68 (69.4) | 0.35 |
| Nausea, n (%) | 60 (33.3) | 27 (32.9) | 33 (33.7) | 0.92 |
| Anorexia, n (%) | 105 (58.3) | 49 (59.8) | 56 (57.1) | 0.72 |
| Itching, n (%) | 59 (32.8) | 40 (48.8) | 19 (19.4) | <0.0001 |
| Muscle Cramping, n (%) | 37 (20.6) | 16 (19.5) | 21 (21.4) | 0.75 |
| SOB, n (%) | 57 (31.7) | 25 (30.5) | 32 (32.7) | 0.76 |
| Edema, n (%) | 133 (73.9) | 60 (73.2) | 73 (74.5) | 0.84 |
| Irritability, n (%) | 1 (0.6) | 0 (0.0) | 1 (1.0) | 1.00 |
| Sleepiness, n (%) | 53 (29.4) | 31 (37.8) | 22 (22.5) | 0.024 |
| Mental status change, n (%) | 5 (2.8) | 2 (2.4) | 3 (3.1) | 1.00 |
| Restless legs, n (%) | 1 (0.6) | 1 (1.2) | 0 (0.0) | 0.46 |
| Change in functional status, n (%) | 9 (5.0) | 4 (4.9) | 5 (5.1) | 1.00 |

**Supplemental Table 3:** Additional clinical data at time of first dialysis

|  | **Total**  **(N=180)** | **Chinese American**  **(N=82)** | **Others (N=98)** | **p-value** | |
| --- | --- | --- | --- | --- | --- |
| Systolic Blood Pressure, mean (SD) | 141.8 (25.8) [N=154] | 141.1 (23.8) [N=75] | 142.4 (27.6) [N=79] | 0.76 | |
| Diastolic Blood Pressure, mean (SD) | 71.1 (15.0) [N=153] | 68.3 (14.3) [N=75] | 73.7 (15.3) [N=78] | 0.025 | |
| Calcium, mean (SD) | 8.6 (0.9)  [N=152] | 8.7 (0.9) [N=73] | 8.6 (0.9)  [N=79] | 0.85 |  |
| PTH, median [Q1-Q3] | 275 [204-432] [N=62] | 276 [203-450] [N=30] | 264 [204-351] [N=32] | 0.50 |  |
| Serum albumin, mean (SD) | 3.5 (0.6)  [N=129] | 3.3 (0.7) [N=64] | 3.7 (0.5)  [N=65] | 0.0003 |  |
| Phosphorous, mean (SD) | 5.2 [4.5-6.3] [N=151] | 5.2 [4.5-6.4] [N=74] | 5.3 [4.5-6.1] [N=77] | 0.73 |  |
| BUN, mean (SD) | 82.2 (29.1) [N=164] | 82.4 (27.6) [N=79] | 82.0 (30.6) [N=85] | 0.93 |  |
| Hemoglobin, mean (SD) | 9.7 (1.6)  [N=145] | 9.8 (1.4) [N=67] | 9.7 (1.8)  [N=78] | 0.87 |  |
| Bicarbonate, mean (SD) | 21.3 (4.1) [N=165] | 21.7 (3.9) [N=77] | 20.9 (4.3)  [N=88] | 0.25 |  |
| Potassium, median [Q1-Q3] | 4.6 (0.7)  [N=163] | 4.6 (0.7) [N=77] | 4.5 (0.7)  [N=86] | 0.58 |  |
| Creatinine, mean (SD) | 6.3 (3.2)  [N=165] | 6.5 (2.9) [N=78] | 6.1 (3.4)  [N=87] | 0.44 |  |

Supplemental Table 4: Additional linear regression of log-transformed time between first CKD 4 visit and first dialysis

| **Variable** | **Estimate** | **Standard Error** | **p Value** |
| --- | --- | --- | --- |
| Intercept | -1.22 | 0.38 | 0.002 |
| Chinese American | 0.21 | 0.10 | 0.03 |
| Age at eGFR <30 mL/min/1.73m^2^ | 0.002 | 0.004 | 0.56 |
| Female | 0.08 | 0.10 | 0.41 |
| eGFR<30 mL/min/1.73m^2^ | 0.05 | 0.01 | <0.0001 |
| Serum albumin at eGFR <30 mL/min/1.73m^2^ | 0.03* | 0.01 | 0.0002 |

*Effect of a 0.1-unit change in serum albumin

Supplemental Table 5: Additional negative binomial regression of rate of visits between first CKD 4 visit and first dialysis

| **Variable** | **Incidence**  **Rate Ratio** | **95% Confidence Limits** | | **p-value** |
| --- | --- | --- | --- | --- |
| Chinese American | 1.09 | 0.68 | 1.75 | 0.73 |
| Age at eGFR <30 | 0.99 | 0.98 | 1.01 | 0.41 |
| Value of eGFR <30 | 0.96 | 0.92 | 1.00 | 0.03 |
| Female | 0.82 | 0.51 | 1.32 | 0.42 |
| Serum Albumin at eGFR <30 | 0.97* | 0.93 | 1.00 | 0.05 |

*IRR for a 0.1-unit change in serum albumin
